# The AMBU® Aura-i™ Laryngeal Mask and LMA Supreme™: A Randomized Trial of Clinical Performance and Fibreoptic Positioning in Unparalysed, Anaesthetised Patients by Novices

**DOI:** 10.1155/2016/4717061

**Published:** 2016-10-25

**Authors:** Zanahriah Yahaya, Wendy H. Teoh, Nora A. Dintan, Ravi Agrawal

**Affiliations:** ^1^Department of Women's Anaesthesia, KK Women's and Children's Hospital, Singapore; ^2^Private Anaesthesia Practice, Wendy Teoh Pte. Ltd., Singapore

## Abstract

*Introduction*. Manikin studies' data cannot accurately be extrapolated to real-life scenarios and inherent differences in design and materials of newer products may affect their clinical performance.* Methods*. Hence, we compared the AMBU® Aura-i™ and LMA Supreme™ in this randomized trial involving 100 ASA 1-2 unparalysed anaesthetised patients undergoing minor gynaecological surgery. Investigators had <20 Aura-i insertions. Primary outcome was time to achieve effective ventilation and secondarily insertion parameters, oropharyngeal leak pressures (OLP), fibreoptic positioning, and pharyngeal morbidity. The position of the Ambu Aura-i was evaluated with the Ascope; the fiberoptic view of the glottis was scored on a five-point scale.* Results*. 43 (86%) AMBU Aura-i and 44 (88%) LMA Supremes were successfully inserted on first attempt (*p* = 0.59), with similar ease (*p* = 0.79), and comparable times to first capnogram, mean (SD) 18.2 (6.0) versus 17.3 (6.4) sec, *p* = 0.9. The Aura-i needed significantly less volume of air to inflate its cuff to 60 cmH_2_O on the manometer, 17.7 (3.5) versus 23.1 (4.4) mL, *p* < 0.001. Both devices exhibited similar OLP, Aura-i versus LMA Supreme, mean (SD) 28.8 (7.1) versus 27.3 (5.3) cmH_2_O, *p* = 0.24. There was no difference in ease of insertion or adjustment manoeuvres to aid ventilation. 90% of patients had good positioning of Aura-i on fibreoptic check, yielding a view of the vocal cords and epiglottis. In 5 patients (10%), the vocal cords were not seen, but ventilatory function was unaffected.* Conclusions*. The Aura-i handled well in novices hands, with comparable times to insert and establish ventilation, similar leak pressures, and successful first attempt insertion rates compared to the LMA Supreme.

## 1. Introduction

The AMBU Aura-i™ (AMBU A/S, Ballerup Denmark) is a polyvinyl chloride, MRI compatible, single-use supraglottic airway device introduced clinically in 2010. It comprises an airway tube with a compliant preformed 90° angle bend designed to mimic the natural curvature of the orohypopharyngeal cavity, a soft rounded tip, 0.4 mm thin cuff, and a bowl devoid of aperture bars, thereby allowing direct endotracheal (ET) intubation. Apart from functioning as a routine supraglottic ventilatory device, this feature makes it potentially useful in difficult airways as a conduit for tracheal intubation and airway-exchange techniques with a fibreoptic scope.

The Laryngeal Mask Supreme™ (LMA-S™, Laryngeal Mask Company, Singapore) on the other hand was introduced in 2007 and is already in widespread clinical use. It has similar characteristics to the AMBU Aura-i™ (single use device, preformed curve and built-in bite block) but is made of stiffer material and differs fundamentally in its design incorporating a gastric drain tube and presence of epiglottic bars. The latter hinders passage of an ET tube and is thus unsuited as a conduit for airway exchange techniques, as passage of even a 4.5 mm fibreoptic bronchoscope is awkward, and the use of an Aintree Intubating Catheter may fail [1].

Supraglottic airways are used prevalently in our ambulatory surgical centre where short minor gynaecological procedures are performed with patients often spontaneously breathing. We postulated that the AMBU Aura-i™ in this setting for routine airway management could confer an added advantage of direct intubation capability should the need arise. This occurs when transvaginal and transcervical myoma resections and hysteroscopic polypectomies are sometimes complicated by uterine perforations, necessitating emergent laparoscopic repair with controlled ventilation and administration of neuromuscular blocking agents. To date, no study has been conducted evaluating how the AMBU Aura-i™ performs compared to the widely used LMA Supreme™.

Due to the inherent differences in design and materials, we aimed to compare the clinical performances of the AMBU Aura-i™ and LMA Supreme™ in spontaneously breathing patients under general anaesthesia. Our primary outcome measure was time taken to achieve effective ventilation (time to first ETCO_2_ establishment). We studied secondary outcomes of ease of insertion, oropharyngeal leak pressures (OLP), fibreoptic position, haemodynamic response, and pharyngeal morbidity (sore throat, blood on device, dysphonia, and dysphagia).

## 2. Methods

The trial was approved by the Singhealth Centralised Institutional Review Board (approval number CIRB 2012-256-D) and registered on the Australian New Zealand Clinical Trials Registry (ANZCTR) [ACTRN 12614000032651]. All patients gave written informed consent.

One hundred ASA I-II patients scheduled for elective minor gynaecological procedures in our tertiary maternity and women's hospital were recruited. We excluded patients of ASA physical status III or IV; BMI > 40 kg/m^2^, those with predicted difficult airway; high risk of regurgitation or aspiration; and respiratory tract pathology (including preoperative sore throat).

Patients were randomly allocated into two groups, “AMBU Aura-i™” or “LMA Supreme™,” using a computer generated random number table. After recruitment, the enrolling investigators opened sealed, opaque envelopes that concealed the group allocation. Patients were blinded to their allocation group. Routine preuse leak tests were performed and the appropriate size of airway was selected according to manufacturer's recommendations. For AMBU Aura-i™, a size 3 was used for patients weighing 30–50 kg, size 4 for 50–70 kg, and size 5 for 70–100 kg. For the LMA Supreme™, a size 3 was used if patients weighed <50 kg, size 4 if 50–90 kg, and size 5 if >90 kg. Both airway devices were lubricated with Aquagel™ and prepared for insertion with the cuff completely deflated.

All patients received a standard general anaesthetic. They were positioned supine on the operating table, with the head resting on a jelly doughnut. Standard monitors consisting of pulse oximetry, electrocardiograph, noninvasive blood pressure, capnograph, inspired oxygen, and volatile agent analysers were applied. Patients were preoxygenated with high flow oxygen for three minutes prior to induction of anaesthesia with intravenous fentanyl 1-2 *μ* kg^−1^ and propofol 2.0–3.0 mg kg^−1^. Upon loss of eye lash reflex and adequate relaxation of the jaw, the device was inserted. The cuff of the devices was inflated with air to attain a cuff pressure of 60 cmH_2_O as measured with a handheld aneroid manometer (Portex® Pressure Gauge, Smiths Medical Intl Ltd, Kent, UK) and the amount of the air needed to inflate the cuff was measured. The appearance of the first square end-tidal carbon dioxide (ETCO_2_) trace denoted successful establishment of effective ventilation. Otherwise, the device was completely removed for another insertion attempt. Three insertion attempts were allowed. Each “attempt” was defined as reinsertion of the airway device into the mouth. We defined “insertion failure” of the device as one comprising >3 unsuccessful attempts or if the entire process of insertion exceeded 120 seconds. This included the time the airway device was removed from the mouth and any bag-mask ventilation in between. In case of failure of both devices, the airway was secured according to the decision of the attending anaesthetist.

After successful placement of the device, oropharyngeal leak pressure (OLP) was then measured by closing the adjustable pressure limiting (APL) valve with a fresh gas flow of 3 L min^−1^, noting the airway pressure at equilibrium or when there was audible air leak from the throat. Maximum pressure allowed was 40 cmH_2_O. The epigastrium was also auscultated when measuring the OLP to detect any air entrainment in the stomach.

The position of the AMBU Aura-i™ was evaluated with the aScope™ 2 (disposable flexible videoscope) by positioning the tip of the aScope™ 1 cm proximal to the airway orifice. The fibreoptic view of the glottis was scored according to an established scoring system as follows: 0: failure to function with no cords seen; 1: cords not seen but function adequate; 2: cords plus anterior epiglottis seen; 3: cords plus posterior epiglottis seen; 4: only cords seen [2]. The airway was considered functioning adequately if the minimal expired tidal volume was 6 mL/kg, peripheral oxygen saturation >95%, CO_2_ <45 mmHg with a respiratory rate of 12–14 min, and a fresh gas flow of 3 L/min without an oropharyngeal leak or gastric insufflation.

It was planned* a priori* that positioning of the LMA Supreme™ would not be checked fibreoptically as its epiglottic fins would hinder passage of a bronchoscope and though not impossible would take longer to perform and interfere logistically with the rapid turnover of minor surgical procedures in the ambulatory centre.

Maintenance of anaesthesia was achieved with oxygen : air mixture in 1-2 MAC sevoflurane with patients spontaneously breathing. Analgesia was provided perioperatively with titrated boluses of fentanyl and either paracetamol or diclofenac suppositories. At the end of surgery, patients were transferred to the recovery area in the supine position with the cuff still inflated. The airway device was removed by either the anaesthetist or the recovery nurse when the patient awakened. The airway device was then inspected for presence of visible blood. Forty-five minutes later, patients were assessed by an independent observer for postoperative sore throat, dysphonia, or dysphagia.

An unblinded observer not involved in the study recorded patient demographics, anthropometric data, duration of surgery, number of insertion attempts, the subjective ease of insertion of the airway device on a 5-point scale (1: easy; 2: not so easy; 3: difficult; 4: very difficult; 5: impossible), time to establish effective ventilation (interval from when the LMA Supreme™ or AMBU Aura-i™ entered the mouth to first ETCO_2_ trace), blood pressure and heart rate every minute for the first five minutes from induction of anaesthesia, and manoeuvers required to optimize positioning or ventilation with the airway devices: adjusting head and neck position, depth of insertion, applying jaw lift, and changing device size, as well as complications of placement (desaturation <95%, gross regurgitation or aspiration (defined as fluid in the ventilation tube), bronchospasm, and mucosal, lip, tongue, or dental injury).

### 2.1. Investigator Proficiency

Apart from the senior author WHLT who supervised all insertions, all investigators were novices in placing the AMBU Aura-i™ with <20 device uses prior to trial commencement but were however proficient LMA Supreme™ users of 3–5-year experience.

### 2.2. Statistics and Sample Size

The primary outcome tested was time to LMA insertion. Secondary outcomes were successful insertion at first attempt, oropharyngeal leak pressure, haemodynamic changes on insertion, and complications of LMA placements. Sample size was based on earlier studies of AMBU and LMA insertion in nonparalysed, anaesthetized patients [3, 4]. Prospective power analysis showed that a sample size of 50 patients per group would be required to detect a 30% difference in the primary outcome at a significance level of 5% and power of 80%.

The following tests were used to compare the data between the two groups: Student's *t*-test for patient demographics and time to insertion; Mann-Whitney *U*-test for Mallampati grading, ease of insertion, and number of attempts; general linear model for mean arterial pressure and heart rate; chi-square/Fisher's exact test for comparison of proportions. All statistical analyses were performed using SPSS 16.0 (IBM) software. Data are presented as mean (standard deviation) or median (interquartile range [range]). A *p* value of <0.05 was considered significant.

## 3. Results

**Figure 1:**
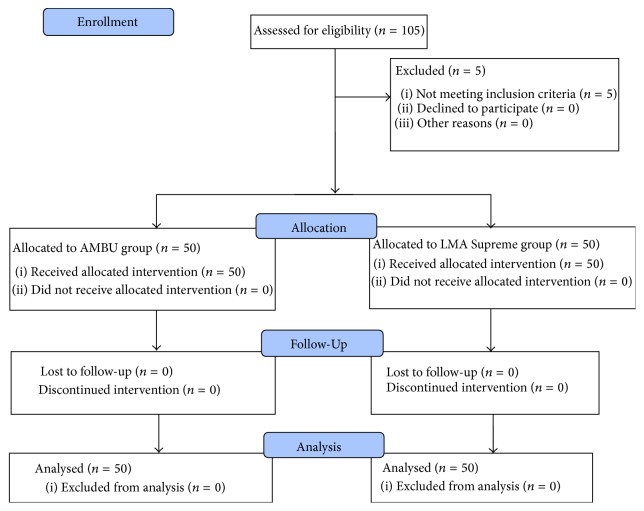
Flow of patients through the study.

Patients were recruited from May 2012 to Dec 2012. One hundred and five patients were assessed for eligibility, five were excluded, and 100 were randomized, allocated to intervention, completed the trial, and were analysed (Figure 1 CONSORT flow diagram). There were no intergroup differences in baseline demographics, anthropometric airway features, type, or duration of surgery (Table 1). Forty-three (86%) AMBU Aura-i™ and 44 (88%) LMA Supremes™ were successfully inserted on first attempt (*p* = 0.59), with similar ease (*p* = 0.79), and comparable times to the first capnograph trace (mean (SD) 18.2 (6.0) versus 17.3 (6.4) sec, *p* = 0.9). The AMBU Aura-i™ needed significantly less volume of air to inflate its cuff to 60 cmH_2_O on the manometer, 17.7 (3.5) versus 23.1 (4.4) mL, *p* < 0.001. Both devices exhibited similar oropharyngeal leak pressures (OLP), AMBU Aura-i™ versus LMA Supreme™, mean (SD) 28.8 (7.1) versus 27.3 (5.3) cmH_2_O, *p* = 0.24. Significantly, eight (16%) AMBU Aura-i™ demonstrated an OLP <20 cmH_2_O compared to only one in the LMA Supreme™ group, *p* = 0.031. There was no difference in ease of insertion or adjustment manoeuvres to aid ventilation. 90% of patients had good positioning of AMBU Aura-i™ on fibreoptic check, yielding a view of the vocal cords and epiglottis. In 5 patients (10%), the vocal cords were not seen, but ventilatory function was adequate (Table 2). No one desaturated or had regurgitation, bronchospasm, ventilatory failure, lip injury, tongue trauma, dental injury, or dysphonia in this trial. Pharyngeal morbidity was insignificant (AMBU Aura-i™ versus LMA Supreme™: dysphagia 2 versus 1 patient, mucosal injury 5 versus 6 patients, and sore throat 7 versus 5 patients) (Table 3). Haemodynamic changes were similar except for the diastolic blood pressure at 5 min (*p* = 0.035), mean arterial pressure at 5 min (*p* = 0.023), and heart rate at 3 min (*p* = 0.033) (Table 4).

## 4. Discussion

Our study found that, in the hands of novices, the Aura-i™ performed comparably to the conventional tried and tested LMA Supreme™ with equally high successful insertion rates on first attempt and overall success, with a similar duration to achieve effective ventilation. On a subjective five-point scale, no insertions were deemed “very difficult” or “impossible,” with a majority (80–88%) categorised as “easy.” Its easy insertion can be attributed to the curvature of the AMBU Aura-i™ mask and airway tube, which was deemed more compliant and bendable compared to the unweilding rigid curvature of the LMA Supreme™. Other authors have reported an equally high successful insertion at first attempt with the AMBU (albeit an older version) compared to the LMA as well, ranging from 83% to 92% [3–5].

The mean (SD) amount of air needed to inflate the AMBU Aura-i™ cuff to 60 cmH_2_O was 17.7 (3.5) mL, approximately 5.4 mL less than needed by the LMA Supreme™. This could be due to inherent differences in the material and although statistically significant was not clinically significant as both devices yielded comparable mean seal pressures of 27-28 cmH_2_O. We did however find oropharyngeal leak pressures of <20 cmH_2_O in 8 AMBU Aura-i™ and only one LMA Supreme™ after uneventful insertion (*p* = 0.03) but it was inconsequential as ventilation was not compromised; surgeries were of short duration and patients not subjected to positive pressure ventilation.

We used the flexible optical AMBU aScope™ 2 to further assess correct placement of the Aura-i™ as it was a new device to us. In 88%, the AMBU Aura-i™ was found aligned in the midline of the airway with complete visualization of the vocal cords, in tandem with either the anterior or the posterior portion of epiglottis. In 10% of patients, the vocal cords were not fully seen but functioned adequately, and in a further 2%, only the cords were seen in our series of patients. Others had previously investigated if nine clinical tests correlated with fibreoptic grade [6]. Presence of resistance at the end of insertion, inability to advance LMA after cuff inflation, and presence of a capnographic trace correlated poorly, whereas the ability to generate an airway pressure of 20 cm water, the ability to ventilate manually, a black line on the LMA in midline, anterior movement of the larynx, outward movement of the LMA on inflation of the cuff, and movements of the reservoir bag with spontaneous breathing were found to correlate well. Of all these, the two clinical tests that best correlated with the fibreoptic grade we described above were the ability to generate an airway pressure of 20 cm water and the ability to ventilate manually [6].

We found the aScope™ 2 to be user-friendly and adequate for performing fibreoptic checks of the airway. We did not encounter any fogging in the fifty cases and despite not having a working channel did not find this to be a shortcoming, as any secretions were easily eradicated with a flick of its tip. The advantages of a disposable flexible scope (its availability, elimination of post-use cleaning procedures and any risk of cross-contamination, repair costs and maintenance) combined with an acceptable clinical performance make the aScope™ 2 a valuable supplement or alternative to other flexible, reusable fibre- and videoscopes.

Previous work on the LMA Supreme™ from our institution found a low postoperative sore throat incidence of 8-9% [7, 8]. Similarly low rates were found in this study, 7/50 (14%) with AMBU Aura-i™ and 5/50 (10%) patients with LMA Supreme™ and no significant differences in oropharyngeal morbidity.

To the best of our knowledge, our study represents the first head-to-head comparison of the ventilatory efficacy of the relatively new Aura-i™ intubating laryngeal mask against the tried and tested LMA Supreme in adults. The Aura-i™'s role as a conduit for elective fibreoptic intubation has been discussed [9] and in children, it performed equally to the air-Q™ [10]. Another report describes an airway rescue with the Aura-i™ in an elderly gentleman after failed direct laryngoscopy. After intubation through the Aura-i™, it was possible to successfully pass a gastric tube behind the Aura-i™ laryngeal mask with its cuff deflated [11]. A simulation study by novice physicians found that chest compressions did not decrease the ventilation and intubation success rates of four intubating supraglottic airway devices, the AURA-i®, air-Q®, i-gel®, and Fastrack® during cardiopulmonary resuscitation [12].

This study had a few limitations. First the patient population was limited to elective patients with normal airway anatomy (all females due to the women's hospital the study took place in) and our results may not be applicable to patients with difficult airways and those with severe retrognathia. Secondly, as with all airway studies, it is impossible to blind the independent observer to the type of airway device used when he/she is performing contemporaneous data collection of airway insertion parameters, and hence potential bias cannot be fully discounted.

Nonetheless this study showed that, in conclusion, the AMBU Aura-i™ handled well in novices hands, with comparable times to insert and establish ventilation with similar successful first attempt insertion rates to the LMA Supreme.

## Figures and Tables

**Table 1:** Patient characteristics. Data are expressed as mean (SD) or numbers of patients (*n*) and percentages (%).

	AMBU Aura-i	LMA Supreme
	(*n* = 50)	(*n* = 50)
Age, years	41.1 (11.7)	37.9 (11.1)
Height, cm	158.1 (5.7)	157.1 (5.6)
Weight, kg	61.2 (14.2)	60.0 (11.6)
Body mass index, kg m^−2^	24.5 (5.5)	24.4 (6.0)
ASA class I/II, *n* (%)	28/22 (56.0%/44.0%)	39/11 (78.0%/22.0%)
Mallampati class 1/2/3/4 (*n*) (%)	14/22/13/1	21/14/10/5
	(28.0%/44.0%/26.0%/2.0%)	(42.0%/28.0%/20.0%/10%)
Thyromental distance; *n* (%)		
<6.5 cm	14 (28%)	15 (30.0%)
>6.5 cm	36 (72%)	35 (70.0%)
Sternomental distance, *n* (%)		
<12.5 cm	8 (16.0%)	12 (24.0%)
>12.5 cm	42 (84.0%)	38 (76.0%)
Interincisor distance, *n* (%)		
<4 cm	17 (34.0%)	13 (26.0%)
>4 cm	33 (66.0%)	37 (74.0%)
Ability to prognath, *n* (%)		
Yes : No	40 (80.0%) : 10 (20.0%)	41 (82.0%) : 9 (18.0%)
Head/neck movement, *n* (%)		
Normal >90° : abnormal <90°	49 (98.0%) : 1 (2.0%)	50 (100%) : 0
Duration of surgery, minutes	25.9 (12.0)	26.2 (8.4)
Type of surgery, *n* (%)		
Uterine dilatation and curettage, hysteroscopy	22 (44.0%)	21 (42.0%)
Myomectomy	0	1 (2.0%)
Evacuation of uterus	3 (6.0%)	9 (18.0%)
Termination of pregnancy	10 (20.0%)	11 (22.0%)
Others	15 (30.0%)	8 (16.0%)

**Table 2:** Airway insertion characteristics and performance of airway devices. Values are expressed as mean (SD) or numbers of patients (*n*) and percentages (%).

	AMBU Aura-i (*n* = 50)	LMA Supreme (*n* = 50)	*p* value
Size of airway used: 3/4/5	11/39/0	7/43/0	0.436
Number of insertion attempts, *n* (%)			
1	43 (86.0%)	44 (88.0%)	0.591
2	5 (10.0%)	6 (12.0%)	
3	2 (4.0%)	0	
Reported ease of insertion, *n* (%)			
1 = easy	40 (80.0%)	42 (88.0%)	0.792
2 = not so easy	6 (12.0%)	6 (12.0%)	
3 = difficult	4 (8.0%)	2 (4.0%)	
4 = very difficult	0	0	
5 = impossible	0	0	
Time to successful airway insertion, s^†^	18.2 (6.0)	17.3 (6.4)	0.900
Cuff volume at 60 cmH_2_O, mL	17.7 (3.5)	23.1 (4.4)	<0.001
Manoeuvres to optimize ventilation			
None	37 (74.0%)	42 (84.0%)	0.784
Adjusting head/neck position	4 (8.0%)	3 (6.0%)	
Changing depth of insertion	1 (2.0%)	0	
Applying jaw lift	1 (2.0%)	1 (2.0%)	
Reinserting the device	7 (4.0%)	4 (8.0%)	
Changing device size	0	0	
Oropharyngeal leak pressure, cmH_2_O	28.8 (7.1)	27.2 (5.3)	0.240
Leak pressure <20 cmH_2_O, *n* (%)	8 (16.0%)	1 (2.0%)	0.031
Air leak into stomach at OLP			
Yes : No	4 (8.0%) : 46 (92.0%)	1 (2.0%) : 49 (98.0%)	0.362
Fiberoptic view			
0 = cannot function, no cord seen	0	NA	
1 = cords not seen but function adequate	5 (10.0%)		
2 = cords plus anterior epiglottis seen	34 (68.0%)		
3 = cords plus posterior epiglottis seen	10 (20.0%)		
4 = only cords seen	1 (2.0%)		

^†^Defined as time from insertion of airway device into patient's mouth to the first end-tidal carbon dioxide trace.

**Table 3:** Complications of placement. Values are expressed in mean (SD) or numbers of patients (*n*) and percentages (%).

	AMBU Aura-i (*n* = 50)	LMA Supreme (*n* = 50)	*p* value
Complications of placement, *n* (%)			
Yes : None	13 (26.0%) : 37 (74.0%)	10 (20.0%) : 40 (80.0%)	0.635
Complications of placement			
Desaturation (SpO_2_ < 95%)	0	0	
Gross regurgitation/aspiration	0	0	
Bronchospasm	0	0	
Difficulty in ventilation	0	0	
Lip injury	0	0	
Tongue trauma	0	0	
Mucosal injury	5	6	1.000
Post-op sore throat	7	5	0.760
Dysphonia/hoarse voice	0	0	
Dysphagia	2	1	1.000
Dental injury	0	0	

**Table 4:** Haemodynamic parameters. Values are expressed as mean (SD) or numbers of patients (*n*) and percentages (%).

	AMBU Aura-i (*n* = 50)	LMA Supreme (*n* = 50)	*p* value
Systolic blood pressure, mmHg			
0 minutes	124.5 (22.2)	121.0 (18.1)	0.387
1 minute	111.1 (22.1)	111.3 (18.9)	0.969
2 minutes	103.1 (16.2)	102.5 (18.3)	0.858
3 minutes	100.0 (16.6)	100.8 (17.4)	0.814
4 minutes	96.2 (13.3)	101.1 (15.7)	0.095
5 minutes	96.5 (15.6)	100.3 (20.8)	0.312
Diastolic blood pressure, mmHg			
0 minutes	68.9 (14.5)	69.5 (14.4)	0.836
1 minute	64.3 (14.7)	64.0 (14.3)	0.918
2 minutes	59.2 (13.2)	57.2 (16.0)	0.492
3 minutes	57.7 (15.2)	58.1 (13.4)	0.889
4 minutes	54.2 (12.4)	58.0 (13.4)	0.144
5 minutes	53.2 (13.5)	59.5 (15.7)	0.035^*∗*^
Mean artery pressure, mmHg			
0 minutes	83.1 (13.9)	83.9 (14.7)	0.77
1 minute	77.1 (14.8)	77.0 (14.9)	0.957
2 minutes	71.2 (13.1)	69.7 (16.8)	0.634
3 minutes	70.3 (13.8)	70.1 (14.0)	0.937
4 minutes	63.8 (13.8)	69.5 (14.1)	0.044^*∗*^
5 minutes	65.0 (12.3)	71.2 (14.6)	0.023^*∗*^
Heart rate, beat per minute			
0 minutes	79.1 (12.8)	76.8 (11.3)	0.336
1 minute	76.6 (14.1)	76.9 (13.1)	0.918
2 minutes	73.0 (12.6)	75.6 (12.8)	0.308
3 minutes	69.0 (11.1)	73.9 (11.5)	0.033^*∗*^
4 minutes	68.7 (11.5)	72.3 (13.4)	0.147
5 minutes	68.4 (14.1)	71.9 (13.0)	0.198

^*∗*^Significant  *p* value.
